# Supplementary material for: Group-level cooperation in chimpanzees is shaped by strong social ties
Source: Nat Commun. 2021 Jan 22;12:539. doi: 10.1038/s41467-020-20709-9 (PMC7822919; doi:10.1038/s41467-020-20709-9)
Supplement: Supplementary file 2 — Description of Additional Supplementary Files [file 41467_2020_20709_MOESM2_ESM.pdf]

Description of Additional Supplementary Files

File Name: Supplementary Data 1
Description: An excel file detailing the data used to fit the ‘female participation’ and ‘male participation’ models.

File Name: Supplementary Code 1
Description: The R code used to fit the ‘female participation’ and ‘male participation’ models.
