# Supplementary material for: Group-level cooperation in chimpanzees is shaped by strong social ties
Source: Nat Commun. 2021 Jan 22;12:539. doi: 10.1038/s41467-020-20709-9 (PMC7822919; doi:10.1038/s41467-020-20709-9)
Supplement: Supplementary file 5 — Reporting Summary [file 41467_2020_20709_MOESM5_ESM.pdf]

nature research

Corresponding author(s): Liran Samuni
Last updated by author(s): Dec 5, 2020

Reporting Summary

Nature Research wishes to improve the reproducibility of the work that we publish. This form provides structure for consistency and transparency in reporting. For further information on Nature Research policies, see Authors & Referees and the Editorial Policy Checklist .

Statistics

For all statistical analyses, confirm that the following items are present in the figure legend, table legend, main text, or Methods section.

n/a	Confirmed	
☐	☒	The exact sample size (*n*) for each experimental group/condition, given as a discrete number and unit of measurement
☐	☒	A statement on whether measurements were taken from distinct samples or whether the same sample was measured repeatedly
☐	☒	The statistical test(s) used AND whether they are one- or two-sided<br>*Only common tests should be described solely by name; describe more complex techniques in the Methods section.*
☐	☒	A description of all covariates tested
☐	☒	A description of any assumptions or corrections, such as tests of normality and adjustment for multiple comparisons
☐	☒	A full description of the statistical parameters including central tendency (e.g. means) or other basic estimates (e.g. regression coefficient) AND variation (e.g. standard deviation) or associated estimates of uncertainty (e.g. confidence intervals)
☐	☒	For null hypothesis testing, the test statistic (e.g. *F*, *t*, *r*) with confidence intervals, effect sizes, degrees of freedom and *P* value noted<br>*Give P values as exact values whenever suitable.*
☒	☐	For Bayesian analysis, information on the choice of priors and Markov chain Monte Carlo settings
☒	☐	For hierarchical and complex designs, identification of the appropriate level for tests and full reporting of outcomes
☐	☒	Estimates of effect sizes (e.g. Cohen's *d*, Pearson's *r*), indicating how they were calculated

Our web collection on statistics for biologists contains articles on many of the points above.

Software and code

Policy information about availability of computer code

Data collection	OR
Data analysis	Data processing and analysis was conducted using R (version 4.0.2), using the packages 'lme4' (version 1.1-25), 'car' (version 3.0-10), and 'MuMIn' (version 1.43.17).

For manuscripts utilizing custom algorithms or software that are central to the research but not yet described in published literature, software must be made available to editors/reviewers. We strongly encourage code deposition in a community repository (e.g. GitHub). See the Nature Research guidelines for submitting code & software for further information.

Data

Policy information about availability of data

All manuscripts must include a data availability statement. This statement should provide the following information, where applicable:

- Accession codes, unique identifiers, or web links for publicly available datasets
- A list of figures that have associated raw data
- A description of any restrictions on data availability

The datasets analyzed in the current study are available as Supplementary Data 1

Field-specific reporting

Please select the one below that is the best fit for your research. If you are not sure, read the appropriate sections before making your selection.

☐ Life sciences ☒ Behavioural & social sciences ☐ Ecological, evolutionary & environmental sciences

Behavioural & social sciences study design

All studies must disclose on these points even when the disclosure is negative.

Study description	This paper includes quantitative data collection procedure of behavioral observations of male and female wild chimpanzees at the Taï National Park, Côte d'Ivoire.
Research sample	Research was held with the Taï Chimpanzee Project (TCP) and the Primatology Department of the Max Planck Institute for Evolutionary Anthropology, at the Taï National Park. We followed three fully-habituated neighbouring social groups, North, South, and East and observed every adult individual of those groups, totaling 36 adult male and 75 adult female chimpanzees. As we intended to study chimpanzee participation in intergroup encounters we only included individuals above 12yrs of age as those are the individuals that make independent participation decisions in encounters. The three groups are considered to be representative of the Taï National Park chimpanzee population.
Sampling strategy	We collected all-day focal animal sampling of adult (>12 years) males and females year round over 58 cumulative observation years. During focal follows, whenever we witnessed an intergroup encounter we switched the data collection protocol to the collection of all occurrence data. As this is a non-invasive study on wild living animals, we did not control the occurrence of the intergroup encounters but instead documented those events whenever they occurred naturally. In addition to data on intergroup encounters, we recorded all demographic changes in our study groups including births, deaths and migration events. Every adult individual in each of the group was followed ca. once per month to achieve a balanced dataset across individuals over time. In all statistical analyses we included the random effect of individual identity with random slopes, to account for within and between individual differences.
Data collection	Focal follow data was collected using pen and paper by a team of experienced observers. To ensure that the data collected was comparable across observers, inter-observer reliability tests were conducted annually. The number of observers following each group varied between 1-4 observers per day, each following a different chimpanzee subject. All observers switched to the collection of all occurrence data during cases of intergroup encounters. None of the observers were aware of the hypotheses or predictions of the current study.
Timing	The data used in this study was collected between the years: North group- 1990-2018; South group- 1999-2018; East group- 2007-2018.
Data exclusions	To investigate participation decisions in intergroup encounters, we must focus the analysis only on data collected during intergroup encounter events that involved an active approach response from the in-group as otherwise no voluntary participation is made. Accordingly, intergroup encounter events without an active participation were not included in the analyses. We provide all information regarding excluded cases within the manuscript main text.
Non-participation	This study includes data collected over several decades from three groups of wild living chimpanzees. Due to natural fluctuations in the groups' demography (e.g., birth, death, migration) not all individuals were followed for the entire course of the study.
Randomization	As data was collected on individuals belonging to three naturally occurring social groups, the allocation to groups in the analytical design were done according to the natural setting of group membership, and group was added as a fixed effect in the statistical analyses.

Reporting for specific materials, systems and methods

We require information from authors about some types of materials, experimental systems and methods used in many studies. Here, indicate whether each material, system or method listed is relevant to your study. If you are not sure if a list item applies to your research, read the appropriate section before selecting a response.

Materials & experimental systems

n/a	Involved in the study	
☒	☐	Antibodies
☒	☐	Eukaryotic cell lines
☒	☐	Palaeontology
☐	☒	Animals and other organisms
☒	☐	Human research participants
☒	☐	Clinical data

Methods

n/a	Involved in the study	
☒	☐	ChIP-seq
☒	☐	Flow cytometry
☒	☐	MRI-based neuroimaging

Animals and other organisms

Policy information about studies involving animals; ARRIVE guidelines recommended for reporting animal research

Laboratory animals	The study did not involve laboratory animals
Wild animals	We collected observational data on 36 male (aged between 12-46yrs) and 75 female (aged between 12-53yrs) wild chimpanzees (Pan troglodytes verus). All methods were non-invasive.

Field-collected samples	This study did not involve samples collected in the field. However, in the analyses we used a pre-existing genetic dataset of the Taï chimpanzee population, identified from fecal sample extracts.
Ethics oversight	This study complied with the ethics policy of the Max Planck Society and was approved by the Ministries of Research and Environment of Côte d'Ivoire, and Office Ivoirien des Parcs et Réserves.

Note that full information on the approval of the study protocol must also be provided in the manuscript.
